# Tuberculosis disease and infection among household contacts of bacteriologically confirmed vs unconfirmed tuberculosis in Cambodia: considerations for optimising contact investigation and preventive treatment

**DOI:** 10.1080/07853890.2026.2714685

**Published:** 2026-08-19

**Authors:** Sovanvorleak Tep, Sovannary Tuot, Sokleaph Cheng, Socheatraksmey Ung, Bertrand Guillard, Saren Menh, Sok Chamreun Choub, Chan Yuda Huot, Laurence Borand, Greg J. Fox, Alvin Kuo Jing Teo

**Affiliations:** ^a^ KHANA and KHANA Centre for Population Health Research, Phnom Penh, Cambodia; ^b^ Department of Community and Global Health, Graduate School of Medicine, The University of Tokyo, Tokyo, Japan; ^c^ Faculty of Social Sciences and Humanity, Royal University of Phnom Penh, Phnom Penh, Cambodia; ^d^ Medical Biology Laboratory, Institut Pasteur du Cambodge, Phnom Penh, Cambodia; ^e^ National Centre for Tuberculosis and Leprosy Control (CENAT), Phnom Penh, Cambodia; ^f^ Clinical Research Group, Epidemiology and Public Health Unit, Institut Pasteur in Cambodia, Phnom Penh, Cambodia; ^g^ Center for Tuberculosis Research, Division of Infectious Diseases, Johns Hopkins University School of Medicine, Baltimore, MD, USA; ^h^ Faculty of Medicine and Health, University of Sydney, Sydney, NSW, Australia; ^i^ University of Sydney Infectious Diseases Institute (Sydney ID), Sydney, NSW, Australia; ^j^ Saw Swee Hock School of Public Health, National University of Singapore and National University Health System, Singapore, Singapore

**Keywords:** Asymptomatic TB, contact investigation, household contacts, IGRA, TB infection, TB preventive treatment (TPT), tuberculosis

## Abstract

**Background:**

This study aimed to describe the yield of tuberculosis (TB) disease among household contacts (HHC) of individuals with bacteriologically confirmed and unconfirmed pulmonary TB, to estimate the prevalence of TB infection using Interferon Gamma Release Assay (IGRA), and to identify factors associated with IGRA positivity.

**Methods:**

We conducted a pragmatic, cross-sectional study in five operational districts between February 2023 and April 2024. HHC of persons with pulmonary TB in the preceding two years were screened for TB. Among eligible contacts (aged ≥5 years and HIV negative) at two purposively selected sites (one urban and one rural), blood samples were collected for IGRA using QuantiFERON^®^-TB Gold Plus. Modified Poisson regression with robust variance estimation was used to identify factors independently associated with IGRA positivity.

**Results:**

Among 864 HHC, 26 were diagnosed with TB disease: 2.7 per 100 (95% CI 1.4–4.6) among contacts of bacteriologically confirmed TB and 3.4 per 100 (95% CI 1.9–5.6) among contacts of bacteriologically unconfirmed TB. More than half were asymptomatic (37.5% bacteriologically confirmed, 66.7% bacteriologically unconfirmed). Of 453 valid IGRA results, 21.2% (95% CI 17.5–25.2) were positive. IGRA positivity was associated with older age, urban residence, and being contacts of bacteriologically confirmed TB.

**Conclusion:**

In these selected programmatic settings, limiting contact investigation to bacteriologically confirmed TB and relying on symptom screening may result in missed cases. Without infection testing, most HHC offered TPT may not require it, while others with asymptomatic TB disease may inappropriately receive preventive treatment.

## Background

Every year, more than 10 million people fall ill with tuberculosis (TB) and a million die from it, making TB the leading infectious cause of morbidity and mortality globally [1]. Approximately 40% of people with TB remain undiagnosed and unreported [1]. In Cambodia, TB remains endemic [2], with an estimated incidence of 272 per 100,000 population in 2024 [1]. The recently-concluded third national TB prevalence survey in Cambodia estimated that TB prevalence among individuals aged 15 years and older was 546 per 100,000 population (95% uncertainty interval: 461–631). Approximately 48,000 Cambodians fell ill with TB in 2024, yet 30% were unreached, undiagnosed, and unnotified [1]. Similarly, worldwide, TB preventive treatment (TPT) uptake among eligible contacts of bacteriologically confirmed TB has remained <50% in recent years [1].

Contacts of people with TB are at high risk of developing TB [3]. Current national guidelines and the World Health Organisation’s (WHO) recommendations for TB contact investigations focus on individuals with bacteriologically confirmed pulmonary and drug-resistant TB [4,5]. Despite this guidance, contact investigation (CI) is not systematically implemented in Cambodia [6,7]. Furthermore, TB symptoms are commonly used as an entry point into the screening algorithm. Given that more than 50% of people with TB in the community may be asymptomatic [8], this leads to missed opportunities for diagnosis and linkage to care. While active case finding and contact investigation are key strategies for case detection and provision of TB preventive treatment (TPT) [5], critical gaps remain: 1) the yield of TB among household contacts (HHCs) of bacteriologically unconfirmed TB is insufficiently characterised. Although less infectious than bacteriologically confirmed cases, persons with bacteriologically unconfirmed TB (also known as ‘clinically diagnosed’ TB) could still transmit the infection to family members and community members [9]. However, this population has been systematically excluded from TB contact investigations in most settings. 2) TB infection rates among contacts older than 5 years who are HIV-negative in Cambodia, groups currently eligible for TPT, are unknown. Closing these gaps by identifying the subgroups to be screened during contact investigation, their characteristics, and the infection status of those eligible for TPT is essential for strengthening case detection and refining TPT strategies.

This study aimed to 1) determine the yield of active TB among HHC of people with bacteriologically confirmed and unconfirmed pulmonary TB, 2) estimate the prevalence of TB infection among HHCs of people with bacteriologically confirmed and unconfirmed pulmonary TB using Interferon Gamma Release Assay (IGRA), and 3) explore factors associated with IGRA positivity among HHCs of people with pulmonary TB.

## Methods

### Study design and settings

We conducted a pragmatic, cross-sectional study in five operational districts (OD), namely Bor Keo (Ratanakiri province), Koh Sotin (Kampong Cham province), Prek Pnov (Phnom Penh), Sre Ambel (Koh Kong province), and Baray Santuk (Kampong Thom province) between February 2023 and April 2024. This study was nested within the Community Mobilisation Initiatives to End Tuberculosis (COMMIT), a 5-year community-based project led by KHANA, a local non-governmental organisation in Cambodia, that aimed to address TB diagnosis, treatment, and prevention needs, as well as stigma and discrimination [10]. COMMIT integrated CI and community active case-finding (ACF) interventions in selected sites, thereby providing an opportunity to evaluate the yield of CI among close contacts (household members and neighbours) of persons with pulmonary TB.

### Study population

We screened HHC (of all ages) of persons with pulmonary TB (bacteriologically confirmed and unconfirmed) from the health centres’ TB registers in the preceding two years. The NTP considers the greatest risk of progression to TB disease to be within the first 2 years of infection, thereby targeting close contacts of index cases identified in the preceding 2 years. We also recruited HHC of persons with pulmonary TB identified during community-based TB active case finding. We included all HHCs who agreed to undergo the screening and testing procedures. We excluded those who refused to participate and unable/unwilling to give informed consent. Pregnant women and children under 10 years old were not eligible for chest X-ray screening; however, sputum samples were collected if they were symptomatic (fever, weight loss, cough, or night sweats). For infection testing, HHC aged ≥5 years and HIV negative (per WHO and national guidelines) were eligible; HHC aged <5 years and people living with HIV were not tested for TB infection because policies for TPT initiation do not require infection testing in these groups [11].

### Sample size and selection

We included all HHC screened during the study period in this evaluation. No sample size was pre-specified. IGRA testing was conducted at two purposively selected sites (1 urban and 1 rural) due to the limited availability of test kits (*n* = 465). Half of the tests were assigned to test eligible participants in Baray–Santuk (rural), and the other half to those in Prek Pnov (urban). The ODs were chosen based on practical considerations and feasibility, specifically in areas where active case finding interventions were already in place. HHC at the selected sites were consecutively recruited until the quota for each district was met.

### Screening, testing, and laboratory procedures

#### TB screening and contact investigation

**Figure 1.**
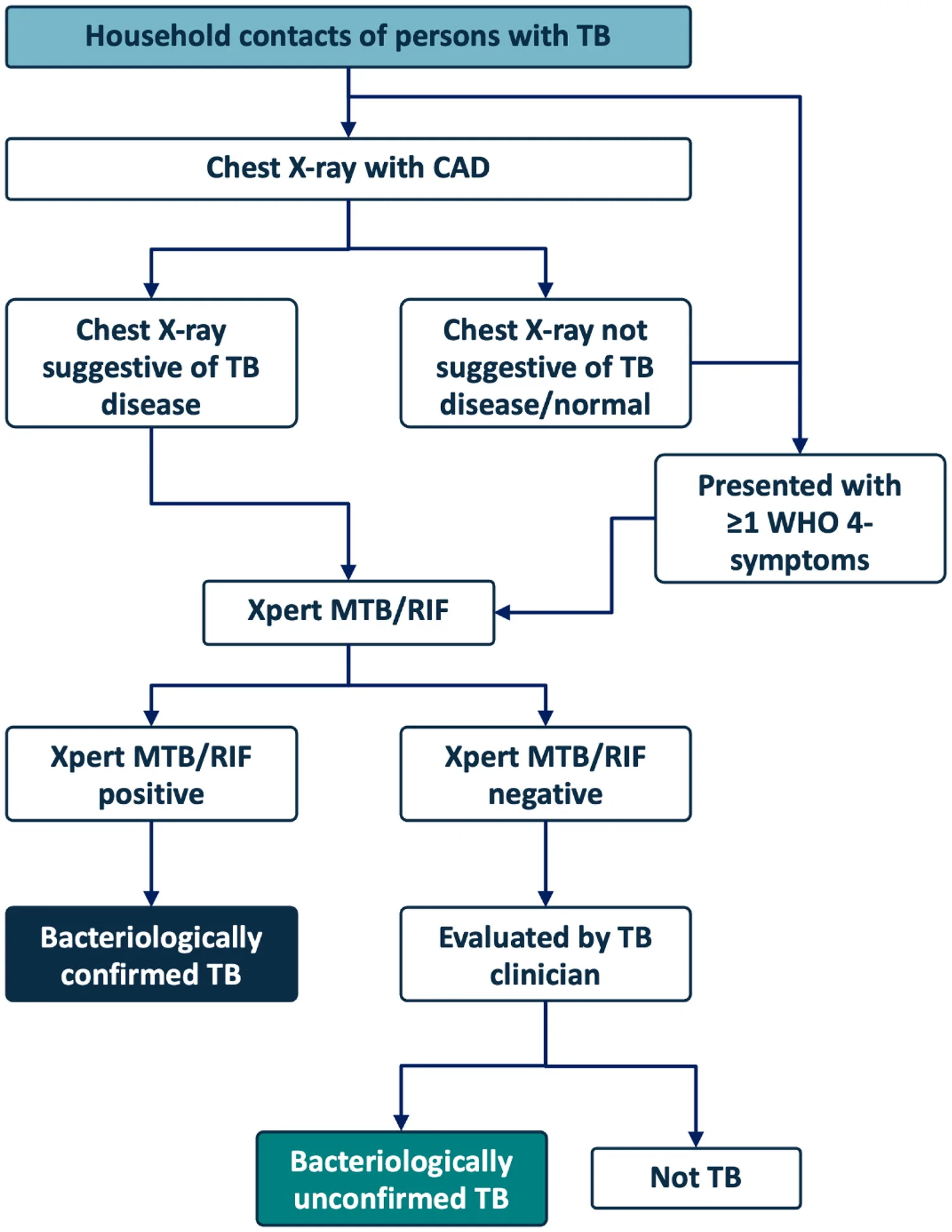
**Community-based tuberculosis disease screening and diagnostic algorithm in Cambodia.** WHO 4-symptoms screen includes cough, fever, night sweats, and weight loss. Chest X-ray suggestive of TB was defined CAD4TB score ≥60 or clinician assessment of the chest X-ray as suggestive of TB irrespective of CAD4TB score. TB, tuberculosis; CAD, computer-aided detection; WHO, World Health Organization; MTB/RIF, *Mycobacterium tuberculosis* and Rifampicin.

All participants were screened for TB disease using digital chest X-ray equipped with computer-aided detection (CAD4TB), regardless of TB symptoms (Figure 1). Individuals with TB symptoms (WHO four-symptoms screen pre-diagnosis) or an abnormal chest X-ray suggestive of TB (CAD4TB score ≥ 60 or clinician assessment of the chest X-ray as suggestive of TB irrespective of CAD4TB score) provided one spontaneous sputum sample for GeneXpert MTB/RIF testing. Chest X-ray CAD outputs were validated by trained personnel but the images were not reviewed by a blinded radiologist. All results were reviewed by a clinician to determine their diagnosis, and treatment for TB was initiated when appropriate. A subset of participants also underwent TB infection testing using the QuantiFERON^®^-TB Gold Plus (IGRA). In line with WHO recommendations and national guidelines [12,13], Cambodia offers TPT to all eligible HHC of bacteriologically confirmed TB, even without TB testing. All HHCs of bacteriologically confirmed TB without evidence of TB disease were considered for TPT, in accordance with national guidelines and routine practice, irrespective of IGRA results. Due to operational constraints, 253 participants underwent upfront IGRA testing; however, IGRA results did not alter TB disease screening, and participants with a positive result subsequently underwent the same screening algorithm as all other household contacts.

#### Blood sample collection, processing, and testing

IGRA testing was performed at the Medical Biology Laboratory (MBL) of the Institut Pasteur du Cambodge (IPC). The IPC-MBL is accredited with NF EN ISO 15189: 2022 by COFRAC and has robust internal and external quality control standards, including TB-related assays.

Peripheral venous blood (5 ml) was collected by trained personnel into lithium heparin tubes. The tubes were transported to IPC-MBL at 2–8 °C. Blood was transferred into four QFT-Plus blood collection tubes (Nil, TB1, TB2, and Mitogen; QIAGEN GmbH, Germany) with 1 mL dispensed into each tube. Tubes were immediately shaken 10 times to ensure the antigens on the tube walls dissolved. The aliquoted QFT-Plus blood collection tubes were incubated upright at 37 °C (±1 °C) for 16–24 h. After incubation, tubes were centrifuged at 2000–3000*g* for 15 min to separate plasma. Plasma samples were stored in a centrifuged blood collection tube at 2–8 °C for up to 28 days prior to analysis.

Interferon-γ (IFN-γ) concentrations were measured using the QFT-Plus ELISA (QIAGEN GmbH, Germany) according to the manufacturer’s instructions. Plasma samples and standards were equilibrated to room temperature (*22 °C* ± 5 °C) prior to testing. A standard curve was prepared using recombinant human IFN-γ standards (8.0 IU/mL) and serial dilutions (4.0, 1.0, 0.25, and 0 IU/mL). Fifty microliters of plasma and conjugate were added to microplate wells pre-coated with anti-human IFN-γ monoclonal antibody, incubated for 120 ± 5 min, washed, and developed with enzyme substrate solution for 30 min. The reaction was stopped with sulfuric acid, and optical density was read at 450 nm with a 620–650 nm reference filter. IFN-γ concentrations were calculated using a log–log standard curve, and results were interpreted per QFT-Plus criteria.

### Study outcomes

In this study, we described 1) the number, proportion, and key characteristics of persons with TB (all forms, including bacteriologically confirmed and unconfirmed) identified per 100 HHCs screened and tested; 2) the key characteristics of HHCs who received an IGRA test and their IGRA results, and 3) the factors associated with IGRA positivity among HHCs of people with pulmonary TB.

Bacteriologically confirmed TB was defined as a positive Xpert result for *M. tuberculosis*. Bacteriologically unconfirmed TB (also known as clinically diagnosed TB) was defined as a diagnosis based on a negative sputum test on Xpert, together with either symptoms suggestive of TB, a chest X-ray suggestive of TB, or both. Chest X-rays were categorised as normal; consistent with TB (parenchymal changes typical of active pulmonary TB disease, including cavitary lesions and consolidations); abnormal, TB-related (any abnormality attributed to TB); or features of previous TB (fibrotic/calcified changes indicative of historical infection without current radiological signs of metabolic activity), based on the CAD4TB score ≥60. IGRA results were generated automatically by the laboratory and interpreted independently, blinded to participants’ contact and clinical status.

We defined key characteristics as follows: sex (male/female), age (in years), currently smoking (no/yes), diabetes (self-reported, no/yes), HIV (self-reported, no/yes), and previously treated for TB (self-reported, no/yes). Symptomatic contacts were those reporting at least one WHO four-symptom screen (cough, fever, night sweats, or weight loss) [5], while those without any of these four symptoms were classified as asymptomatic. Trained personnel assessed the presence of a BCG scar as evidence of prior vaccination.

### Statistical analysis

Categorical variables were summarised as frequencies and percentages, and continuous variables as medians with interquartile ranges. Group comparisons used the chi-square test for categorical variables and the Wilcoxon rank-sum test for continuous variables. We limited the description of IGRA positivity to HHC, valid results, and those without TB disease. We presented the 95% confidence interval (CI) for the proportions using the Clopper–Pearson exact method. The number needed to screen and test to detect one person with all forms of TB (NNS) was calculated by dividing the total number screened by the number of people with TB identified.

The analysis of risk factors associated with IGRA positivity was exploratory. We used modified Poisson regression with robust variance estimation and accounted for clustering at the household level to estimate adjusted *prevalence ratios (aPRs) and 95% CIs*. Variables included in multivariable models were selected based on epidemiological relevance and covariates with *p* ≤ 0.10 in bivariate analyses, with model selection guided by the Akaike Information Criterion. An aPR > 1 indicated a higher likelihood of IGRA positivity. Statistical significance was defined as a two-tailed *p* < 0.05. Analyses were performed using Stata version 19 (StataCorp, College Station, TX, USA).

## Results

### TB disease screening among HHCs of persons with pulmonary TB

**Figure 2.**
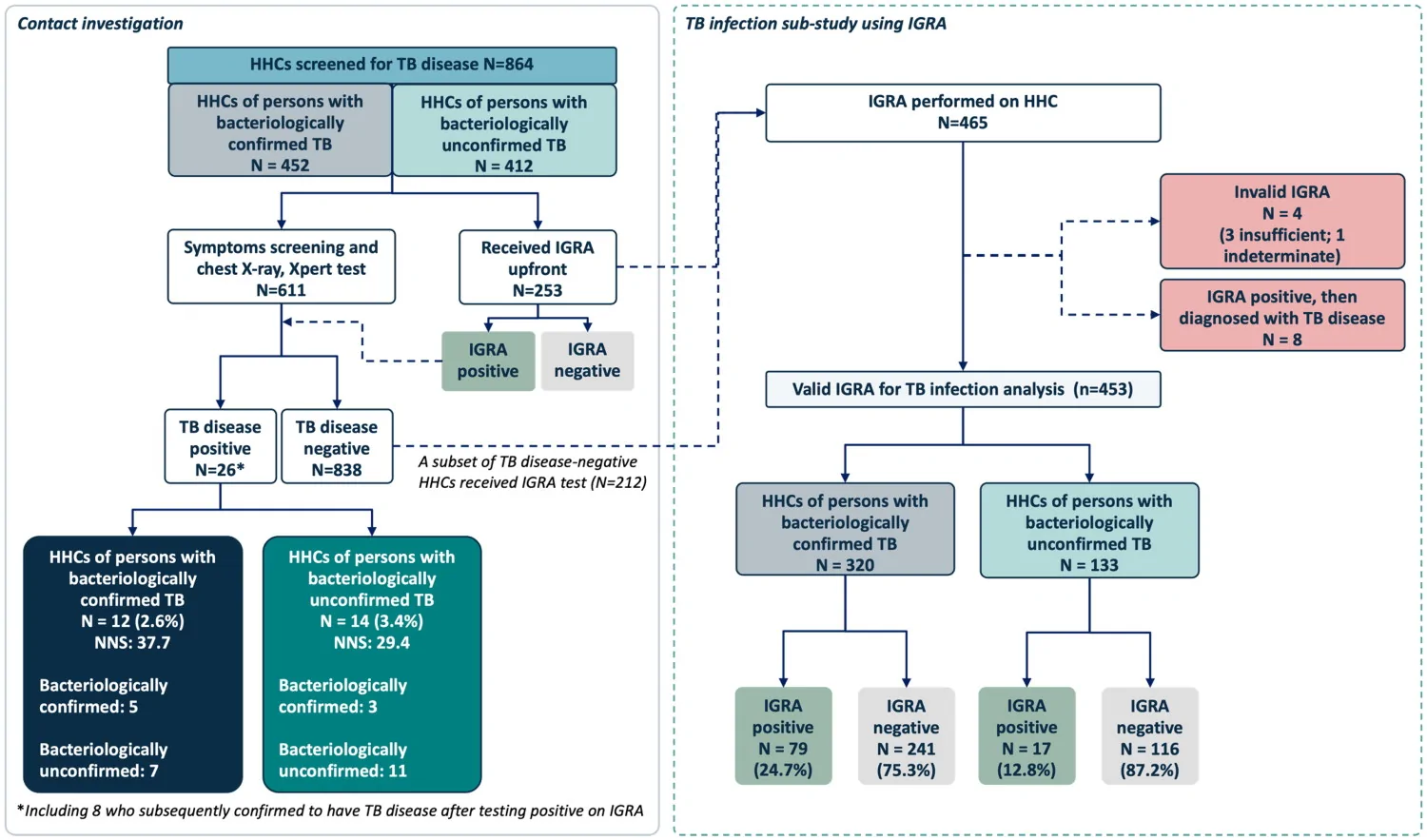
**Flow diagram of household contact investigation and IGRA sub-study.** TB, tuberculosis; HHC, household contact; IGRA, interferon-gamma release assay; NNS, number needed to screen and test.

Among 864 HHCs screened, 452 were contacts of persons with bacteriologically confirmed TB and 412 were contacts of persons with bacteriologically unconfirmed TB (Table 1; Figure 2). The cohort was predominantly female, with a similar distribution across contact groups. The median age was 53 years (IQR 34–67 years) and 58 years (IQR 39–68 years), respectively. Current smoking and diabetes were infrequent. No participants reported HIV infection.

**Table 1.** Characteristics of household contacts screened for TB.

	HHCs of persons with bacteriologically confirmed TB	HHCs of persons with bacteriologically unconfirmed TB
Total	452	412
Sex, *n* (%)		
Female	289 (63.9)	250 (60.7)
Male	163 (36.1)	162 (39.3)
Age, median (IQR)	53 (34–67)	58 (39–68)
Smoking, *n* (%)*		
No	208/223 (93.3)	267/284 (94.0)
Yes	15/223 (6.7)	17/284 (6.0)
Diabetes, *n* (%)*		
Yes	9/223 (4.0)	19/284 (6.7)
No	214/223 (96.0)	265/284 (93.3)
HIV, *n* (%)*		
Yes	0/223 (0.0)	0/284 (0.0)
No	223/223 (100.0)	284/284 (100.0)
Previously treated for TB, *n* (%)*		
Yes	11/223 (4.9)	13/284 (4.6)
No	212/223 (95.1)	271/284 (95.4)
Pulmonary TB disease, *n* (%)		
No	440 (97.4)	398 (96.6)
Yes	12 (2.6)	14 (3.4)
Number needed to screen and test to detect one person with all-forms TB (NNS)	37.7	29.4

TB; tuberculosis, IQR; interquartile range.

*Excludes missing values: Denominators differed for smoking, diabetes, HIV and previous TB. Percentages were calculated among those with available data.

Among the HHCs, a total of 26 persons with active TB were identified (3.0 per 100 screened [95% CI 2.0–4.4), comprising 8 cases of bacteriologically confirmed pulmonary TB and 18 cases of bacteriologically unconfirmed pulmonary TB. TB was diagnosed in 2.7 per 100 (95% CI 1.4–4.6) contacts living with a person with bacteriologically confirmed TB (12/452) and 3.4 per 100 (95% CI 1.9–5.6) contacts living with a person with bacteriologically unconfirmed TB (14/412). The number needed to screen and test to detect one person with any form of TB was 37.7 (95% CI 21.8–72.5) and 29.4 (95% CI 17.7–53.5), respectively.

### Clinical and diagnostic features of TB among HHC

Among the eight bacteriologically confirmed cases of pulmonary TB among HHCs, 50.0% were female, with a median age of 59.5 years (IQR 51.5–64); 62.5% were symptomatic at screening. Most of the chest radiographs were abnormal, and among the bacteriologically confirmed cases, presumptive TB was most common. In contrast, among bacteriologically unconfirmed pulmonary TB, 33.3% were female, with a median age of 71 years (IQR 63–73); 33.3% were symptomatic. All those with bacteriologically unconfirmed TB had negative sputum GeneXpert results, while 94.4% had abnormal TB findings on chest radiography (Table 2). Among bacteriologically confirmed pulmonary TB cases, 62.5% were HHCs of persons with bacteriologically confirmed TB. Among contacts with bacteriologically unconfirmed pulmonary TB, 38.9% were HHC of an index person with bacteriologically confirmed TB.

**Table 2.** Characteristics of HHCs detected with TB.

	Bacteriologically confirmed pulmonary TB (*n* = 8)	Bacteriologically unconfirmed pulmonary TB (*n* = 18)
Sex, *n* (%)		
Female	4 (50.0)	6 (33.3)
Male	4 (50.0)	12 (66.7)
Age, median (IQR), years	59.5 (51.5–64)	71 (63–73)
Smoking, *n* (%)*		
Yes	1/7 (14.3)	2/10 (20.0)
No	6/7 (85.7)	8/10 (80.0)
Diabetes, *n* (%)*		
Yes	0/7 (0.0)	1/10 (10.0)
No	7/7 (100.0)	9/10 (90.0)
HIV, *n* (%)*^¶^		
Yes	0/7 (0.0)	0/10 (0.0)
No	7/7 (100.0)	10/10 (100.0)
Previously treated for TB, *n* (%)*		
Yes	1/7 (14.3)	0/10 (0.0)
No	6/7 (85.7)	10/10 (100.0)
TB symptoms, *n* (%)^†^		
Symptomatic	5 (62.5)	6 (33.3)
Asymptomatic	3 (37.5)	12 (66.7)
Types of HHCs, *n* (%)		
HHCs of persons with bacteriologically confirmed TB	5 (62.5)	7 (38.9)
HHCs of persons with bacteriologically unconfirmed TB	3 (37.5)	11 (61.1)
GeneXpert MTB/RIF, *n* (%)		
Negative	0 (0.0)	18 (100.0)
MTB detected	5 (62.5)	0 (0.0)
MTB detected trace	0 (0.0)	0 (0.0)
MTB detected RIF resistance indeterminate	3 (37.5)	0 (0.0)
MTB detected RIF resistance detected	0 (0.0)	0 (0.0)
Not available (error, invalid, no results)	0 (0.0)	0 (0.0)
Chest X-ray, *n* (%)^††^		
Normal	0 (0.0)	1 (5.6)
Consistent with TB	2 (25.0)	0 (0.0)
Abnormal TB	4 (50.0)	17 (94.4)
Features of previous TB	1 (12.5)	0 (0.0)
Not done/no information	1 (12.5)	0 (0.0)

TB; tuberculosis, IQR; interquartile range, MTB; Mycobacterium tuberculosis, RR; Rifampicin resistance, RIF; rifampicin, HHCs; household contacts.

*Excludes missing values: Denominators differed for smoking, diabetes, HIV and previous TB. Percentages were calculated among those with available data.

^†^WHO four-symptom screen (cough, fever, night sweats, or weight loss). Considered symptomatic if reported ³1 symptom/s. Those without symptoms were classified as asymptomatic.

^††^Interpreted using computer-aided detection software.

^¶^HIV status was self-reported.

### Testing for TB infection among HHCs of persons with pulmonary TB

Of 453 valid IGRA results (excluding those subsequently confirmed to have TB disease after testing positive on IGRA (*n* = 8), insufficient sample (*n* = 3) and indeterminate results (*n* = 1)), 21.2% (95% CI 17.5–25.2) were positive (Table 3).

**Table 3.** Characteristics of household contacts with a valid IGRA test result.

	QuantiFERON-TB Gold Plus negative (row percents)	QuantiFERON-TB Gold Plus positive (row percents)	*p* value
Total	357	96	
Sex, *n* (%)			0.071
Female	221 (76.2)	69 (23.8)	
Male	136 (83.4)	27 (16.6)	
Age, median (IQR), years	31 (14–47)	42 (18.5–56.5)	0.005
Age groups in year, *n* (%)			0.164*
<15 years	102 (87.2)	15 (12.8)	
15–24 years	47 (82.5)	10 (17.5)	
25–34 years	45 (76.3)	14 (23.7)	
35–44 years	60 (76.9)	18 (23.1)	
45–54 years	34 (73.9)	12 (26.1)	
55–64 years	33 (71.75)	13 (28.3)	
≥65 years	36 (72.0)	14 (28.0)	
Urbanicity, *n* (%)			0.003
Rural	203 (84.2)	38 (15.8)	
Urban	154 (72.6)	58 (27.4)	
Total number of dwellers living in the same household regardless of study participation, median (IQR)	5 (4–7)	5 (4–6)	0.277
Current smoker, *n* (%)			0.535
No	337 (79.1)	89 (20.9)	
Yes	20 (74.1)	7 (25.9)	
Alcohol consumption, *n* (%)			0.581
No	273 (79.8)	69 (20.2)	
Monthly or less	43 (71.7)	17 (28.3)	
2–4 times per month	20 (76.9)	6 (23.1)	
2–3 times per week	9 (90.0)	1 (10.0)	
≥4 times per week	12 (80.0)	3 (20.0)	
BCG scar, *n* (%)			0.078
Yes	196 (82.0)	44 (18.0)	
No/not sure	159 (75.2)	53 (24.8)	
TB diagnosis of the index person, *n* (%)			0.005
Bacteriologically confirmed	241 (75.3)	79 (24.7)	
Bacteriologically unconfirmed	116 (87.2)	17 (12.8)	
	Median IU/mL (range)	Median IU/mL (range)	
TB1-Nil	0.00 (−0.42, 0.33)	0.87 (−0.08, 9.86)	<0.001
TB2-Nil	0.01 (−0.43, 15.85)	0.92 (0.15, 9.86)	<0.001
TB2-Nil-TB1-Nil	0.00 (−0.53, 15.87)	0.01 (−0.67, 9.14)	0.597

Percentages are shown as row percentages; *p* values were calculated using Chi-square test unless otherwise specified.

TB; tuberculosis, IQR; interquartile range, BCG; Bacillus Calmette–Guérin.

*Cochran–Armitage trend test.

IGRA positivity was more frequent in older age groups, and in urban than in rural settings (27.4% vs. 15.8%; *p* = 0.003). IGRA positivity differed by the index person’s TB classification: 24.7% among contacts of bacteriologically confirmed TB vs. 12.8% among contacts of bacteriologically unconfirmed TB (*p* = 0.005). Other behavioural factors (current smoking and alcohol consumption frequency) were not significantly associated with IGRA positivity. The number of household dwellers did not differ between IGRA groups.

In adjusted models, IGRA positivity was associated with older age and was higher among contacts residing in urban settings than in rural settings (aPR 1.80, 95% CI 1.25–2.60; *p* = 0.001). IGRA positivity was lower among contacts of bacteriologically unconfirmed TB relative to contacts of bacteriologically confirmed TB (aPR 0.56, 95% CI 0.35–0.90; *p* = 0.017) (Table 4).

**Table 4.** Factors associated with IGRA positivity.

	Adjusted prevalence ratio (95% confidence interval)	*p* value
Sex		0.115
Male	1.00	
Female	1.37 (0.93–2.00)	
Age in years		
<15	1.00	
15–24	1.29 (0.60–2.77)	0.517
25–34	1.81 (0.96–3.42)	0.069
35–44	1.64 (0.88–3.09)	0.122
45–54	1.82 (0.90–3.71)	0.098
55–64	2.24 (1.23–4.08)	0.009
≥65	2.38 (1.27–4.48)	0.007
Urbanicity		0.007
Rural	1.00	
Urban	1.76 (1.17–2.65)	
TB type of the index person		0.038
Bacteriologically confirmed	1.00	
Bacteriologically unconfirmed	0.55 (0.32–0.97)	

Adjusted prevalence ratios from modified Poisson regression with robust variance estimation clustered by household (*n* = 210 households); the model included sex, age, urbanicity and index-case bacteriological status.

## Discussion

### Household contact investigation and implications for TB case finding

Our evaluation of household contact investigation across five Cambodian districts showed that the proportion of HHC diagnosed with bacteriologically unconfirmed TB was numerically similar to that among HHC diagnosed with bacteriologically confirmed TB. This difference may not be statistically significant due to overlapping confidence intervals, likely reflecting limited statistical power given the small number of TB cases identified. Nevertheless, these findings challenge the prevailing practice of prioritising contact investigation only for HHC of patients with bacteriologically confirmed TB. The current case-finding approach is risk-group-focused and systematically excludes HHCs of bacteriologically unconfirmed TB cases [14]. Consequently, TB among this population is routinely missed. Relying on self-care-seeking not only delays diagnosis and treatment and increases morbidity/mortality but also risks perpetuating transmission within households and communities [15]. Expanding contact investigation to include all HHCs, irrespective of index bacteriological status, is therefore one of the many strategies to closing detection gaps. This is consistent with the possibility that IGRA positivity and disease yields among contacts of bacteriologically unconfirmed index cases may reflect high background community transmission rather than direct transmission from the index case [16]. Accordingly, in high-burden settings, population-wide screening may be more effective for maximising case detection than risk-group-based approaches, such as household contact investigation [17].

Cambodia, like many other high-burden countries, has identified a substantial proportion of asymptomatic TB in the community [18]. In our study, two-thirds of bacteriologically unconfirmed pulmonary TB and one-third of bacteriologically confirmed pulmonary TB were asymptomatic at screening. While symptom-based screening is simple and less expensive to implement, relying on it as the gatekeeper would have overlooked many of these cases [8].

Asymptomatic TB often had radiographic features consistent with TB, underscoring the added value of chest X-ray in TB disease screening. WHO recommends chest X-ray as a screening tool, with a reported sensitivity of 94.7% for detecting bacteriologically confirmed pulmonary TB when any abnormality is considered [5,19]. While highly sensitive and capable of increasing case detection, chest X-ray faces practical challenges: dependence on centralised facilities where chest Xray is available (district or provincial hospitals) and capacity of the radiology department to perform and read X-rays, unsuitability for pregnant individuals, and the possibility of still missing cases because of non-specific radiological features (among people living with HIV patients or young children, for instance) and the high proportion of asymptomatic TB [20–23]. In the absence of an affordable rapid molecular test for universal testing, our findings support integrating sensitive tools, such as chest radiography, into contact investigation and active case-finding strategies. This reinforces the global call to reconsider the role of symptom screening, particularly in the community, and advocate for a symptom–agnostic approach to improve case detection [24,25].

The higher yield and lower NNS observed in households with bacteriologically unconfirmed index cases compared to bacteriologically confirmed households highlight the need to investigate factors contributing to negative bacteriological results, including sputum quality, timing of specimen collection, and pre-analytical handling [26]. Strengthening sputum collection protocols through systematic coached expectoration [27,28] and quality assurance of sputum sample collection could reduce false negatives and improve index case classification, thereby informing contact investigation priorities. Given the challenges of obtaining high-quality sputum [26], non-sputum-based diagnostic tests that have shown promising results and are currently recommended by the WHO should also be considered [29].

### IGRA positivity and TB preventive treatment strategy

Excluding those contacts with TB disease, we found that 21.2% of HHC with valid IGRA results were IGRA-positive. The infection profile among contacts suggests that disease risk is concentrated in older, urban populations and in households of bacteriologically confirmed TB. The IGRA positive rate corresponds with the latest estimates for the WHO Western Pacific Region at 20.7% (95% CI 16.8–24.5) [30], but is significantly lower than previously estimated at approximately 50% in a 2016 modelling study [31]. This suggests that the risk of TB among HHC may not be markedly higher than in the general community, thereby reinforcing the notion that individuals living in high-burden settings face similarly elevated risk, not only those with a known exposure in the household. However, the higher positivity among contacts of bacteriologically confirmed than of unconfirmed index cases indicates that household exposure to confirmed TB may still confer additional risk.

The country’s first-ever estimate of TB infection (IGRA positivity) among HHCs has direct implications for TPT policies. Under the current policy, TPT may be given to HHC of all ages without TB infection testing, so long as TB disease has been excluded based on symptoms [12]. Excluding HHC aged <5 and people living with HIV who do not require TB infection testing for initiating TPT [11], our findings indicate that most screened contacts do not have a positive IGRA test for infection. A higher likelihood of IGRA positivity among HHC of bacteriologically confirmed TB and among contacts who were older and urban residents suggests opportunities to prioritise TPT in groups likely to derive the greatest benefit. Moreover, one-third of contacts of bacteriologically confirmed TB who had asymptomatic disease would still be eligible for TPT according to current practice, raising concerns about under-treatment of TB disease using a regimen of 1 or 2 antibiotics and contributing to acquired drug resistance. Nonetheless, broader TPT eligibility lowers operational barriers and may confer population-level benefit where infection testing is not feasible. WHO guidance in this area continues to evolve. Our findings, therefore, identify an opportunity to prioritise contacts most likely to benefit, rather than to restrict eligibility.

This study has several limitations. First, this was a pragmatic study embedded within a community-based active case-finding initiative. IGRA testing was confined to purposively selected sites, and due to limited kits, not all household contacts in the selected areas have been screened. However, in practice, contact investigation was not implemented for all index persons with TB due to competing priorities and resource constraints in the public health system. The cross-sectional study design limits causal inference regarding transmission and risk factors within contact groups. Second, small numbers of TB cases meant that the precision of prevalence estimates was limited for most subgroups. Third, most detected cases were clinically diagnosed, and some misclassification is possible given the reliance on clinical and radiographic criteria. However, similar yields were observed when analyses were restricted to bacteriologically confirmed TB and clinically diagnosed TB with radiographic evidence of disease. Any misclassification is unlikely to have differed systematically between contact groups, as the same diagnostic criteria were applied regardless of the index case’s bacteriological status. Furthermore, testing only one spontaneously expectorated sputum sample may have contributed to the lower sensitivity of bacteriological detection. Nevertheless, all diagnoses were reviewed by clinicians in accordance with standard practice. While CXR interpretation was standardised using computer-aided detection and TB clinician interpretation, it was not reviewed and recategorised by a blinded radiologist. Therefore, misclassification might have occurred. Fourth, IGRA testing was purposively limited to two districts where community active case finding was already established, rather than by probability sampling. This limits the generalisability of infection prevalence estimates to the wider Cambodian population of household contacts. Nevertheless, the observed positivity is consistent with pooled regional estimates, which supports the plausibility of the estimate. Fifth, the observed association between urban residence and IGRA positivity may reflect district-level factors such as local TB transmission, healthcare access, and population density, rather than true urban-rural differences in TB exposure, and should be interpreted accordingly. Sixth, in the absence of community comparators and molecular linkage data, our study estimates the yield of TB disease and infection among household contacts rather than transmission attributable to the index case. Because up to 2 years could elapse between index notification and contact screening, TB and IGRA positivity cannot be fully attributed to the household index case. IGRA positivity may also be affected by reversion over time, which would lower the measured rate, even as prolonged exposure could raise it. Seventh, missingness in selected variables and reliance on self-report (e.g. diabetes, smoking, HIV) might have introduced measurement bias. Co-morbidities were self-reported and not verified against medical records. Misclassification cannot be excluded. Therefore, findings on IGRA correlates should be considered exploratory. Lastly, index persons with TB were not enrolled, so characteristics beyond bacteriological status were unavailable.

## Conclusion

In these selected programmatic settings, limiting CI to HHC of bacteriologically confirmed TB and relying on symptom screening as the primary gatekeeper may miss TB cases. Without TB infection testing, most HHCs offered TPT under national guidelines did not have evidence of infection and may not actually need it, while a small number of contacts with asymptomatic TB disease could receive preventive treatment instead of curative therapy. Expanding CI to all HHC, implementing a more sensitive screening algorithm, and refining TPT eligibility criteria are practical steps to close detection gaps and strengthen TPT strategy, alongside efforts to improve TPT uptake and coverage.

## Data Availability

Data may be made available upon reasonable request to the corresponding author and with approval from the ethics committee.
